# Prognostic impact of preoperative skeletal muscle change from diagnosis to surgery in patients with perihilar cholangiocarcinoma

**DOI:** 10.1002/ags3.12644

**Published:** 2022-12-11

**Authors:** Koki Hayashi, Yuta Abe, Minoru Kitago, Hiroshi Yagi, Yasushi Hasegawa, Shutaro Hori, Masayuki Tanaka, Yutaka Nakano, Yuko Kitagawa

**Affiliations:** ^1^ Department of Surgery Keio University School of Medicine Tokyo Japan

**Keywords:** cholangiocarcinoma, hilar, perihilar, sarcopenia, skeletal muscle mass

## Abstract

**Background:**

Loss of skeletal muscle mass is a prognostic factor after surgery for gastrointestinal cancers. The treatment for perihilar cholangiocarcinoma (PHC) is a highly invasive surgery. Biliary drainage and portal vein embolization, which can prolong the preoperative waiting time (PWT), are often required before surgery. Assuming that the skeletal muscle mass can change during PWT, we investigated the clinical effect of skeletal muscle change on surgical outcomes of PHC.

**Methods:**

We retrospectively reviewed the medical records of 89 patients who underwent curative surgery for PHC from January 2013 to December 2019. We defined the psoas muscle area (PMA) at the third lumbar vertebra as the skeletal muscle mass. The PMA just before surgery was divided by that at the time of diagnosis, and we defined it as the rate of change of PMA (CPMA). Patients were divided into two groups according to CPMA: wasting (n = 44, below the median CPMA) and no‐change (n = 45, above the median CPMA).

**Results:**

The median PWT was 63 d, and CPMA was 96.1%. The median recurrence‐free survival and overall survival were significantly shorter in the wasting group than in the no‐change group (8.0 vs 33.2 mo, *P =* 0.001 and 14.2 vs 48.7 mo, *P <* 0.001, respectively). Multivariate analysis revealed that histological differentiation, R1 resection, lymph node metastasis, and preoperative skeletal muscle wasting were independent prognostic factors of PHC.

**Conclusion:**

This study suggests that preoperative skeletal muscle wasting in patients with PHC has a negative effect on survival outcomes.

## Graphical abstract

We investigated the correlation between changes in skeletal muscle mass during the preoperative waiting time and surgical outcomes of patients with perihilar cholangiocarcinoma. Preoperative skeletal muscle wasting had a negative effect on cancer recurrence and survival.

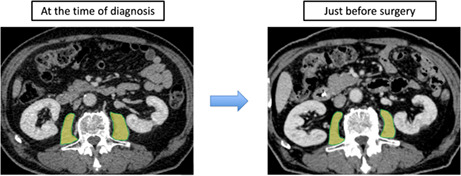

## 1 INTRODUCTION

Perihilar cholangiocarcinoma (PHC) is one of the most aggressive malignancies, and its only potentially curative treatment is aggressive surgical resection, such as major hepatectomy with extrahepatic bile duct resection.^1^, ^2^, ^3^ Before surgery, patients with PHC often require biliary drainage for obstructive jaundice and portal vein embolization to enlarge the remaining liver, and neoadjuvant chemotherapy can be adopted for advanced PHC. Thus, the preoperative waiting time (PWT) from diagnosis to surgery is often longer for PHC than it is for other malignancies.^4^, ^5^

Several studies have shown that sarcopenia, or the loss of skeletal muscle mass, is associated with poor short‐ and long‐term outcomes in PHC.^6^ The onset of sarcopenia can have multiple causes, including immobility, altered endocrine functions, and chronic inflammatory diseases (including malignancy).^7^ Furthermore, rehabilitation and nutritional enhancement before surgery may affect sarcopenia status.^8^, ^9^

Most previous reports assessed patients' sarcopenia status only once before surgery.^10^, ^11^ However, in patients with PHC the sarcopenia status may change throughout the PWT due to cholangitis or other factors, and evaluation at a single point is considered insufficient to thoroughly assess the sarcopenia status of such patients. Thus, research assessing preoperative skeletal muscle change in patients with PHC and its effects on short‐ and long‐term outcomes is necessary.

We aimed to investigate the effect of preoperative skeletal muscle change during PWT on short‐ and long‐term survival outcomes of patients who underwent PHC resection.

## 2 METHODS

### 2.1 Study design and patient population

**FIGURE 1:**
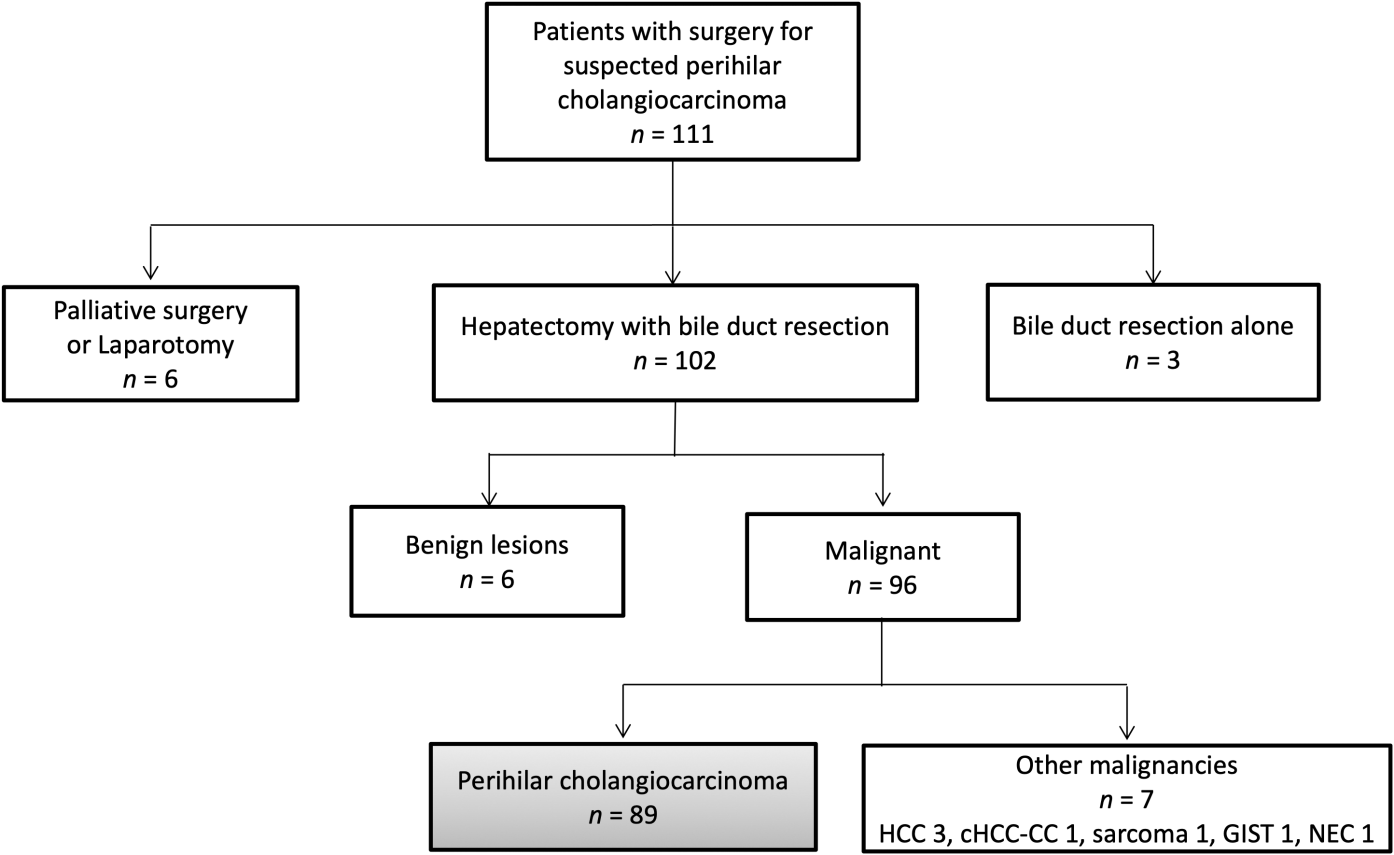
Flow chart of all patients who underwent surgery for suspected perihilar cholangiocarcinoma. HCC, hepatocellular carcinoma; cHCC‐CC, combined hepatocellular and cholangiocarcinoma; GIST, gastrointestinal stromal tumor; NEC, neuroendocrine carcinoma

We retrospectively reviewed the medical records of 111 patients with resectable PHC who underwent surgery at Keio University Hospital (Tokyo, Japan) between January 2013 and December 2019. Of these, six patients who underwent palliative surgery or laparotomy due to distant metastasis or local cancer extension, three who underwent bile duct resection alone, six diagnosed with benign lesions, and seven diagnosed with other malignancies were excluded from the study. Finally, 89 patients who were pathologically diagnosed with PHC were included in the analysis (Figure 1).

Study variables included patient characteristics, laboratory results, tumor characteristics, details of the surgery, complications, and overall survival and recurrence data. Inflammation‐based prognostic scores, which indicate the nutritional status and predict prognosis, such as the prognostic nutritional index (PNI), controlling nutritional status (CONUT), Modified Glasgow Prognostic Score (mGPS), neutrophil–lymphocyte ratio (NLR), and platelet–lymphocyte ratio (PLR), were calculated.^12^, ^13^, ^14^, ^15^, ^16^ Laboratory results obtained from the patients 1–3 d before surgery were used to determine these prognostic scores. Pathologic examination of the tumors was performed according to the 8th edition of the Union for International Cancer Control tumor‐node‐metastasis classification system.^17^

This study was approved by the Ethics Committee of the Keio University School of Medicine (Approval number: 20120443).

### 2.2 Image analysis and definition of preoperative skeletal muscle wasting

**FIGURE 2:**
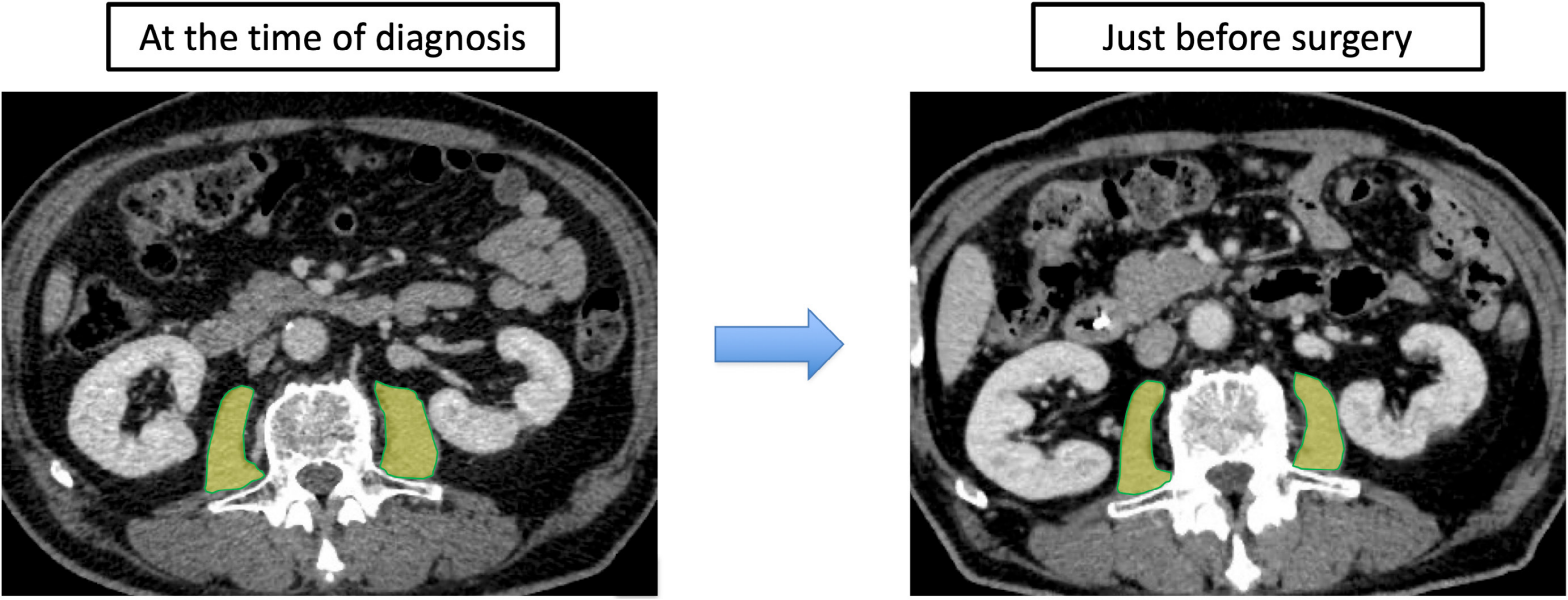
Measurement of the psoas muscle area (PMA) at the time of diagnosis and just before surgery. The bilateral psoas muscle cross‐sectional computed tomography images at the middle‐level of the third lumbar vertebra was measured using SYNAPSE VINCENT software. The border of the bilateral psoas muscle was manually outlined and quantified using Hounsfield unit thresholds (−29 to +150). The PMA just before surgery was divided by that at the time of diagnosis, and it was defined as the rate of change of PMA (CPMA)

All patients underwent abdominal/pelvic computed tomography (CT) at least twice: at the time of cancer diagnosis and just before surgery. The cross‐sectional areas of the right and left psoas muscles (psoas muscle area: PMA) at the middle level of the third lumbar vertebra were measured using SYNAPSE VINCENT software (Fujifilm Co., Tokyo, Japan). The border of the psoas muscle was manually outlined and quantified using a Hounsfield unit threshold from −29 to +150.^18^ The PMA value just before surgery was divided by that at the time of cancer diagnosis, and the resulting value was defined as the rate of change in PMA (CPMA) (Figure 2). According to the median CPMA, patients were divided into the preoperative skeletal muscle wasting and no‐change groups. The clinicopathological factors, as well as the short‐ and long‐term outcomes of the two groups, were compared. The measured PMA was normalized according to height using the following equation: normalized PMA, defined as the psoas muscle mass index (PMI, cm^2^/m^2^) = measured PMA (cm^2^)/height (m^2^). The cutoff level of sarcopenia in PMI value was 6.36 cm^2^/m^2^ for males and 3.92 cm^2^/m^2^ for females.^19^ Obesity was defined as body mass index (BMI) ≥ 25 kg^2^/m^2^.^20^ Sarcopenic obesity was defined as patients with both sarcopenia and obesity.

### 2.3 Preoperative management and surgical procedures

For patients who experienced jaundice before surgery, appropriate biliary drainage, either by endoscopic nasobiliary drainage, endoscopic biliary stent, or percutaneous transhepatic biliary drainage, was performed. If the remnant liver volume was less than 40%, portal vein embolization was performed 2–4 wk before surgery. We considered neoadjuvant chemotherapy for patients who could not undergo R0 resection due to severe local progression.

All surgical procedures were performed after total bilirubin concentrations in the serum decreased to <2 mg/dl. In most cases, patients underwent radical resection of the tumor, which consisted of hilar resection with en‐bloc (extended) hemi‐hepatectomy including the caudate lobe, excision of the portal vein bifurcation when involved, and complete lymphadenectomy of the hepatoduodenal ligament. Combined pancreatoduodenectomy was performed in the following conditions: (1) diffusely infiltrating tumor of the whole extrahepatic bile duct, (2) downward superficial spreading, or (3) bulky nodal metastasis of the pancreatoduodenal region. Vascular resections were carried out only when the vessel adhered to and could not be freed from the tumor during skeletonization resection of the hepatoduodenal ligament. For biliary reconstruction, end‐to‐side anastomoses of the segmental ducts and a Roux‐ex‐Y jejunal loop were constructed. In cases of hepato‐pancreato‐duodenectomy, reconstruction was performed according to the Child method with an end‐to‐side pancreatojejunostomy.

### 2.4 Definitions of complications and follow‐up

Postoperative complications were scored using the Clavien–Dindo classification system.^21^ A major complication was defined as Clavien–Dindo grade III or higher within 30 d after surgery. Posthepatectomy liver failure was defined as increased international normalized ratio value (or need for clotting factors to maintain a normal international normalized ratio) and increased plasma bilirubin levels on or 5 d after the operation affecting clinical management (defined by the International Study Group of Liver Surgery as grade B or C).^22^ Postoperative mortality was defined as death related to surgery. Clinical follow‐up was performed routinely every 3 mo until the second y after surgery and every 6 mo until July 2021 or death. Laboratory tests and follow‐up CT scans were performed in the first 6 mo and later as necessary to detect recurrence. Adjuvant chemotherapy was considered for patients at pathological stage II or higher and for those with a performance status of 0 or 1.

### 2.5 Statistical analysis

All analyses were performed using SPSS software (v. 26; IBM Corp., Armonk, NY, USA). Comparisons were performed using Student's *t*‐test or the Mann–Whitney test for continuous variables and a chi‐squared test or Fisher's exact test for categorical variables. Categorical data were reported as number and frequency (%), and continuous data were reported as median and interquartile range (IQR). The cutoff value of prognostic scores was determined as in previous reports.^16^, ^23^, ^24^ The overall survival (OS) and recurrence‐free survival (RFS) rates were calculated using the Kaplan–Meier method and compared using a log‐rank test, except for patients who died related to surgery. OS was calculated from the date of surgery to the date of death or the most recent follow‐up, and we defined the patients who died of other causes with no evidence of recurrence as censored cases. RFS was measured from the date of surgery to the date of disease recurrence. We performed univariate analyses using Cox proportional hazard regression models to select variables likely related to OS and RFS among all candidate clinicopathological or operation‐related factors. Owing to the limited number of observed events, factors with a *P*‐value <0.05 in the univariate analysis were used in the multivariate analysis with Cox proportional hazard regression models to determine the independent prognostic factors. All results with two‐tailed *P‐*values <0.05 were considered statistically significant.

## 3 RESULTS

### 3.1 Clinicopathological characteristics according to CPMA

The median time from diagnosis to surgery was 63 (IQR: 49–108) d. The median CPMA was 96.1% (IQR: 91.1%–101.2%). According to the median CPMA, we divided the 89 patients into two groups: 45 patients in the skeletal muscle no‐change group and 44 patients in the skeletal muscle wasting group. Table 1 shows comparisons of clinicopathological characteristics between the two groups. We found that the PNI (*P =* 0.042) and CONUT (*P =* 0.020) were significantly worse in the skeletal muscle wasting group compared to the skeletal muscle no‐change group. However, there was no significant difference in preoperative management, such as biliary drainage, percutaneous transhepatic portal vein embolization (PTPE), and neoadjuvant therapy, or the number of days from diagnosis to surgery between the two groups. The presence of sarcopenia or sarcopenic obesity at the time of diagnosis and just before surgery also did not differ significantly. Considering pathological factors, moderate/poor tumor differentiation was significantly common in the skeletal muscle wasting group (*P =* 0.024), while other factors displayed no significant difference.

**TABLE 1:** Patients' clinicopathological characteristics according to change in psoas muscle area

	Preoperative skeletal muscle no‐change (n = 45)	Preoperative skeletal muscle wasting (n = 44)	*P‐*value
A: Summary of patient characteristics			
Male sex, n (%)	27 (60)	28 (64)	0.724
Age, y (IQR)	72 (65–77)	73 (65–77)	0.847
BMI, kg/m^2^ (IQR)	22 (19–23)	21 (19–24)	0.657
ICGR15, % (IQR)	7.8 (5.9–11.6)	7.3 (5.6–11.9)	0.919
CEA, U/ml (IQR)	2.4 (1.5–4.4)	2.3 (1.7–3.3)	0.514
CA19‐9, U/ml (IQR)	45 (24–120)	46 (21–146)	0.831
PNI (IQR)	44 (41–46)	41 (38–45)	0.042
CONUT normal/mild/moderate/severe	12/28/5/0	11/17/15/1	0.020
NLR (IQR)	2.5 (2.0–3.7)	2.8 (2.0–5.2)	0.434
PLR (IQR)	180 (136–249)	176 (139–259)	0.937
mGPS (0/1/2)	30/9/6	20/11/13	0.092
Preoperative biliary drainage +, n (%)	35 (78)	37 (84)	0.449
Preoperative cholangitis +, n (%)	21 (47)	21 (48)	0.920
Preoperative portal vein embolization +, n (%)	24 (53)	24 (55)	0.909
Neoadjuvant therapy +, n (%)	14 (31)	10 (23)	0.373
Sarcopenia at the time of diagnosis, n (%)	31 (71)	25 (57)	0.184
Obesity at the time of diagnosis, n (%)	6 (13)	9 (21)	0.370
Sarcopenic obesity at the time of diagnosis, n (%)	3 (7)	3 (7)	0.651
Sarcopenia just before surgery, n (%)	31 (69)	32 (73)	0.691
Obesity just before surgery, n (%)	5 (11)	7 (16)	0.508
Sarcopenic obesity just before surgery, n (%)	4 (9)	4 (9)	0.630
Number of d from diagnosis to surgery, d (IQR)	56 (49–113)	64 (48–98)	0.678
B: Summary of pathological factors			
T category^a^, n (%)			0.245
Tis, T1, 2	17 (38)	22 (50)	
T3, 4	28 (62)	22 (50)	
Positive lymph node metastasis, n (%)	20 (44)	27 (61)	0.110
Histologic type, n (%)			0.024
Moderate/poor	27 (60)	36 (82)	
Well, papillary	18 (40)	8 (18)	
Curability, n (%)			0.114
R0^b^	38 (84)	31 (71)	
R1, R2	7 (16)	13 (30)	

Abbreviations: BMI, body mass index; CA19‐9, carbohydrate antigen 19–9; CEA, carcinoembryonic antigen; CONUT, controlling nutritional status; ICGR15, indocyanine green retention rate at 15 min; IQR, interquartile range; mGPS, modified Glasgow Prognostic Score; NLR, neutrophil–lymphocyte ratio; PLR, platelet–lymphocyte ratio; PNI, prognostic nutritional index.

^a^ According to the 8th edition of Union for International Cancer Control (UICC) Staging.

^b^ Including patients with positive ductal margin with carcinoma in situ.

### 3.2 Operative factors and short‐term results according to CPMA

In Table 2, comparisons of operative factors and short‐term surgical results are summarized. Combined pancreatoduodenectomy was performed significantly more often in the preoperative skeletal muscle wasting group (*P =* 0.024) compared to the skeletal muscle no‐change group; intraoperative blood loss and postoperative hospital stays were significantly more common in the preoperative skeletal muscle wasting group (*P =* 0.034 and *P =* 0.018, respectively). Additionally, major complications and posthepatectomy liver failure tended to be more common in the preoperative skeletal muscle wasting group compared to the skeletal muscle no‐change group. Finally, mortalities were observed only in the preoperative skeletal muscle wasting group.

**TABLE 2:** Operative factors and short‐term surgical results according to change in psoas muscle area

	Preoperative skeletal muscle no‐change (*n* = 45)	Preoperative skeletal muscle wasting (*n* = 44)	*P‐*value
Type of hepatectomy^a^			0.294
S1, 4, 5, 6, 7, 8, n (%)	1 (2)	1 (2)	
S1, 5, 6, 7, 8, n (%)	16 (36)	19 (46)	
S1, 2, 3, 4, 5, 8, n (%)	7 (16)	6 (14)	
S1, 2, 3, 4, n (%)	16 (36)	13 (30)	
S1, 4, 5, 8/S1, 4, n (%)	4 (9)	5 (11)	
S1, 2, 5, 6, 7, 8, n (%)	1 (2)	0 (0)	
Combined pancreatoduodenectomy, n (%)	4 (9)	12 (27)	0.024
Combined vascular resection, n (%)	22 (49)	20 (46)	0.746
Portal vein resection, n (%)	18 (40)	16 (36)	0.724
Hepatic artery resection, n (%)	10 (22)	10 (23)	0.954
Portal vein and hepatic artery, n (%)	6 (13)	6 (14)	0.967
Operative time, min (IQR)	669 (547–812)	723 (574–874)	0.862
Intraoperative blood loss, ml (IQR)	430 (235–772)	595 (370–981)	0.034
Intraoperative red blood cell transfusion, n (%)	16 (36)	22 (50)	0.168
Preoperative hospital stays, d (IQR)	24 (13–35)	28 (21–42)	0.177
Postoperative hospital stays, d (IQR)	28 (22–44)	40 (26–51)	0.018
Major complications^b^, n (%)	18 (40)	25 (57)	0.112
Liver failure^c^, n (%)	8 (18)	14 (32)	0.125
Mortality^d^, n (%)	0 (0)	3 (7)	0.117

Abbreviation: IQR, interquartile range.

^a^ According to Couinaud hepatic segment.

^b^ Grade III or higher according to the Clavien–Dindo classification.

^c^ Grade B or C according to the criteria of the International Study Group of Liver Surgery.

^d^ Including all deaths related to surgery.

### 3.3 Effects of CPMA on long‐term follow‐up

**FIGURE 3:**
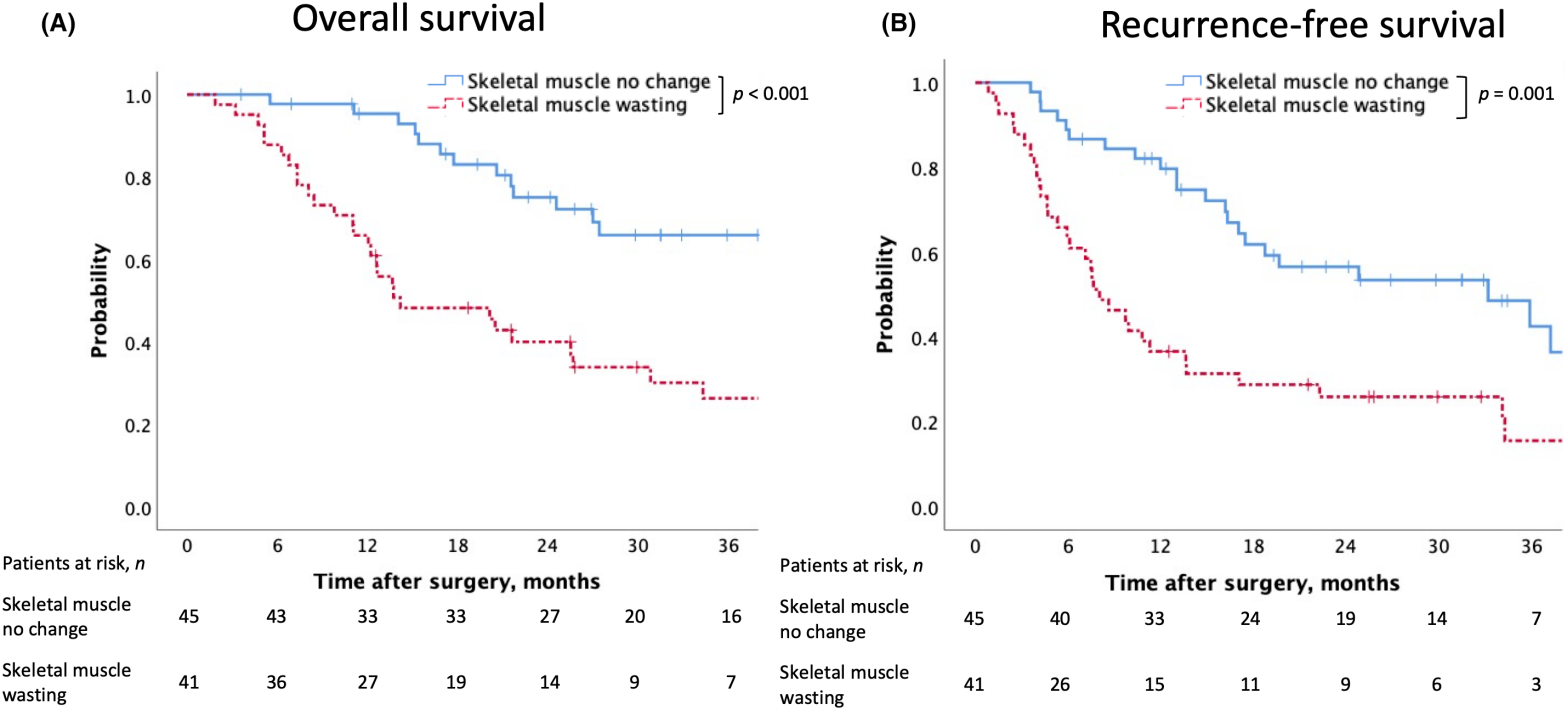
Overall survival (**A**) and recurrence‐free survival (B) rates of patients with resected PHC according to preoperative skeletal muscle change. The patients with preoperative skeletal muscle wasting demonstrated poor overall and recurrence‐free survival (log‐rank *P <* 0.001 and *P =* 0.001, respectively)

The mean time duration of follow‐up for censored cases was 30.7 (IQR: 19.2–50.6) mo. After excluding three cases of operative mortality, the median OS was 14.2 (95% confidence interval [CI] 4.9–23.5) mo in the preoperative skeletal muscle wasting group and 48.7 (95% CI 42.4–55.0) mo in the no‐change group. The 1‐ and 3‐y OS rates were 65.9% and 26.4% in the preoperative skeletal muscle wasting group and 95.3% and 65.9% in the no‐change group, respectively. Moreover, the OS was significantly shorter in the skeletal muscle wasting group according to the log‐rank test (*P <* 0.001, Figure 3a). After excluding three cases of mortality, the median RFS was 8.0 (95% CI 5.2–10.8) mo in the preoperative skeletal muscle wasting group and 33.2 (95% CI 13.8–52.6) mo in the no‐change group. The 1‐ and 3‐y RFS rates were 36.6% and 15.5% in the preoperative skeletal muscle wasting group and 79.7% and 42.5% in the no‐change group, respectively. Like OS, RFS was significantly shorter in the skeletal muscle wasting group according to the log‐rank test (*P =* 0.001, Figure 3b).

### 3.4 Analysis of prognostic factors in PHC

Prognostic factors after resection were analyzed in the 89 patients who underwent curative surgery. In univariate analysis, 8 of 22 possible clinicopathological factors were statistically associated with poor OS (Table 3). Multivariate analysis confirmed skeletal muscle wasting (*P =* 0.016; hazard ratio [HR] 2.67; 95% CI 1.20–5.95), R1/2 resection (*P =* 0.005; HR 3.03; 95% CI 1.40–6.58), positive lymph node metastases (*P =* 0.045; HR 2.41; 95% CI 1.02–5.68), and histologic type (moderate/poor) (*P =* 0.017; HR 11.8; 95% CI 1.55–90.9) to be independent prognostic factors of poor survival. Regarding RFS, 4 of 22 possible clinicopathological factors were statistically associated with the risk of recurrence in univariate analysis (Table 4). Multivariate analysis confirmed skeletal muscle wasting (*P =* 0.029; HR 1.85; 95% CI 1.06–3.23), positive lymph node metastases (*P =* 0.039; HR 1.90; 95% CI 1.03–3.47), and histologic type (moderate/poor) (*P =* 0.005; HR 3.11; 95% CI 1.42–6.80) to be independent prognostic factors of cancer recurrence.

**TABLE 3:** Uni‐ and multivariate Cox regression analyses for overall survival

	Univariate			Multivariate		
	*P‐*value	HR	95% CI	*P‐*value	HR	95% CI
Age (>75 y)	0.237	1.43	0.79–2.60			
BMI (<22)	0.293	1.38	0.76–2.51			
Preoperative jaundice	0.151	1.80	0.81–4.03			
Preoperative cholangitis	0.526	1.20	0.68–2.15			
Preoperative portal vein embolism	0.887	1.04	0.59–1.86			
Preoperative chemo/chemoradiotherapy	0.893	1.05	0.50–1.81			
CA19‐9 (>200)	0.038	2.14	1.04–4.39	0.672	1.23	0.48–3.13
PNI (<45)	0.953	1.02	0.53–1.95			
CONUT (moderate/severe)	0.031	2.16	1.07–4.33	0.440	1.39	0.60–3.21
NLR (>3)	0.587	1.18	0.65–2.16			
PLR (>150)	0.731	1.12	0.59–2.14			
mGPS (1 or 2)	0.725	1.11	0.62–1.99			
Sarcopenia at the time of diagnosis	0.040	1.93	1.03–3.61	0.172	1.92	0.75–4.90
Sarcopenic obesity at the time of diagnosis	0.318	1.69	0.60–4.76			
Sarcopenia just before surgery	0.018	2.29	1.15–4.55	0.233	1.96	0.65–5.99
Sarcopenic obesity just before surgery	0.628	1.59	0.52–2.93			
Skeletal muscle wasting	0.001	2.78	1.52–5.05	0.016	2.67	1.20–5.95
Major complications^a^	0.054	1.76	0.99–3.13			
R1/2 resection	<0.001	3.89	2.11–7.14	0.005	3.03	1.40–6.58
Positive lymph node metastases	0.001	2.82	1.51–5.24	0.045	2.41	1.02–5.68
pT^b^ (≥3)	0.099	1.65	0.91–2.99			
Histologic type (moderate/poor)	0.001	5.10	2.01–12.8	0.017	11.8	1.55–90.9

Abbreviations: BMI, body mass index; CA19‐9, carbohydrate antigen 19–9; CI, confidence interval; CONUT, controlling nutritional status; HR, hazard ratio; mGPS, modified Glasgow Prognostic Score; NLR, neutrophil–lymphocyte ratio; PLR, platelet–lymphocyte ratio; PNI, prognostic nutritional index.

^a^ Grade III or higher according to the Clavien–Dindo classification.

^b^ According to the 8th edition of Union for International Cancer Control (UICC) Staging.

**TABLE 4:** Uni‐ and multivariate Cox regression analyses for recurrence‐free survival

	Univariate			Multivariate		
	*P‐*value	HR	95% CI	*P‐*value	HR	95% CI
Age (>75 y)	0.207	1.42	0.82–2.46			
BMI (<22)	0.312	1.33	0.76–2.33			
Preoperative jaundice	0.265	1.50	0.73–3.08			
Preoperative cholangitis	0.729	1.10	0.65–1.86			
Preoperative portal vein embolism	0.785	1.08	0.63–1.83			
Preoperative chemo/chemoradiotherapy	0.943	1.02	0.56–1.85			
CA19‐9 (>200)	0.684	1.16	0.57–2.39			
PNI (<45)	0.446	1.28	0.68–2.41			
CONUT (moderate/severe)	0.123	1.67	0.87–3.21			
NLR (>3)	0.439	1.25	0.71–2.20			
PLR (>150)	0.708	1.12	0.62–2.03			
mGPS (1 or 2)	0.679	1.12	0.65–1.92			
Sarcopenia at the time of diagnosis	0.247	1.39	0.79–2.44			
Sarcopenic obesity at the time of diagnosis	0.294	1.73	0.62–4.81			
Sarcopenia just before surgery	0.206	1.47	0.81–2.65			
Sarcopenic obesity just before surgery	0.506	1.34	0.57–3.13			
Skeletal muscle wasting	0.001	2.40	1.40–4.12	0.029	1.85	1.06–3.23
Major complications^a^	0.588	1.16	0.68–1.98			
R1/2 resection	<0.001	2.95	1.63–5.35	0.111	1.66	0.89–3.09
Positive lymph node metastases	0.001	2.74	1.55–4.85	0.039	1.90	1.03–3.47
pT^b^ (≥3)	0.057	1.71	0.98–2.95			
Histologic type (moderate/poor)	<0.001	4.20	1.97–9.01	0.005	3.11	1.42–6.80

Abbreviations: BMI, body mass index; CA19‐9, carbohydrate antigen 19–9; CI, confidence interval; CONUT, controlling nutritional status; HR, hazard ratio; mGPS, modified Glasgow Prognostic Score; NLR, neutrophil–lymphocyte ratio; PLR, platelet–lymphocyte ratio; PNI, prognostic nutritional index.

^a^ Grade III or higher according to the Clavien–Dindo classification.

^b^ According to the 8th edition of Union for International Cancer Control (UICC) Staging.

## 4 DISCUSSION

This study demonstrated a median time of 63 d from diagnosis to surgery for patients with PHC and a median CPMA of 96.1%. The PNI and CONUT were significantly worse, and moderate/poor histologic types were more commonly observed in the preoperative skeletal muscle wasting group. However, there were no significant differences in other clinicopathological factors, including sarcopenia, at the time of diagnosis and just before surgery. Preoperative skeletal muscle wasting was significantly associated with long postoperative hospital stays and poor OS and RFS. Furthermore, multivariate analysis showed that, in addition to R1/2 resection, positive lymph node metastases, and moderate/poor histology, preoperative skeletal muscle wasting was also an independent prognostic factor. To the best of our knowledge, this is the first report to focus on the clinical effect of preoperative skeletal muscle change in patients with resectable PHC.

Preoperative sarcopenia was reported to be a predictive factor of postoperative morbidities in several hepatobiliary cancers, including PHC.^6^, ^25^ Similarly, in the present study the preoperative skeletal muscle wasting group tended to have more major complications, including liver failure, and significantly longer postoperative hospital stays than the no‐change group. Additionally, patients in the preoperative skeletal muscle wasting group had a lower PNI and higher CONUT score. This could reflect the malnutrition status in patients with preoperative skeletal muscle wasting, which could have a negative effect on postoperative recovery and tissue repair.^26^ Other reasons for poorer perioperative outcomes in the preoperative skeletal muscle wasting group could be the higher rates of hepato‐pancreato‐duodenectomy and intraoperative blood loss. Although the only observed pathological difference between the two groups was a histologic type, there is possibly more widespread bile duct cancer, which needed to combine pancreatoduodenectomy in the wasting group.

The prognosis after PHC resection depends on several tumor‐specific factors, including lymph node metastasis, differentiation, and resection‐based curability; however, most of these factors can only be determined after the operation. In clinical settings, prognostic factors that can be identified before the operation are more useful. Regarding patient‐specific factors, several retrospective studies have shown that the survival rate of preoperative sarcopenic patients with PHC was significantly lower than that of nonsarcopenic patients.^6^ In this study, preoperative skeletal muscle wasting was one of the significant independent prognostic factors, while other preoperative factors, such as NLR, PLR, mGPS, PNI, and CONUT,^16^, ^17^, ^18^, ^19^, ^20^ were not identified as independent prognostic factors. Skeletal muscle changes may be particularly useful, rather than the inflammation‐based prognostic parameters mentioned above, in patients with PHC who may develop cholangitis during a long PWT.

As in the previous reports,^6^ sarcopenia was significantly associated with OS both at the time of diagnosis and just before surgery in univariate Cox regression analysis. However, skeletal muscle wasting was significantly associated with both OS and RFS in the multivariate Cox regression analysis, while sarcopenia at the time of diagnosis or just before surgery was not. In patients with PHC who have a long PWT, CPMA may be a more useful indicator than PMA at only one point.

Recently, besides sarcopenia, there have been reports that sarcopenic obesity is an important prognostic factor for malignancy.^27^ However, when limited to PHC, while there are some reports of sarcopenic obesity being associated with postoperative complications, there are still few reports about long‐term outcomes.^28^ In the present study, sarcopenic obesity was also not associated with OS and RFS in patients with resected PHC due to univariate Cox regression analysis. Further research is needed on the prognostic impact of sarcopenic obesity in patients with PHC.

The mechanisms by which sarcopenia or skeletal muscle wasting is associated with morbidity and mortality are not fully understood. However, this association could be because more malignant tumors can lead to energy reduction by eliciting an excessive inflammatory response, resulting in loss of muscle mass.^29^ In this study, we observed significantly more poorly differentiated cancers in the preoperative skeletal muscle wasting group than in the skeletal muscle no‐change group. If the sarcopenia is related to tumor malignancy, methods such as the introduction of intense neoadjuvant therapy for patients with preoperative skeletal muscle wasting might be effective in improving prognosis. However, the effectiveness of neoadjuvant therapy for PHC is controversial, and more potent neoadjuvant therapy needs to be established.^30^

On the other hand, enhancing preoperative skeletal muscle mass might improve the prognosis of patients with PHC. Past reports have shown that enhanced physical activity may result in a decreased antiinflammatory response and improved immune function against cancer recurrence.^31^ Several other studies have reported that preoperative rehabilitation or nutritional therapy is effective in decreasing postoperative complications and improving prognosis.^32^, ^33^ Although prospective studies are needed to conclude whether preventing preoperative skeletal muscle mass loss leads to improved prognosis, evaluating the preoperative skeletal muscle changes from diagnosis to surgery may be more useful than observing sarcopenia status at only one point in assessing the effect of interventions such as preoperative rehabilitation or nutritional therapy.

This study had several limitations. First, this study was retrospective in design and included a relatively small number of patients from a single institution. Second, the mean follow‐up period for censored cases was rather short, ie, 30.7 mo. Although the long‐term prognosis of patients with PHC is poor, a longer observation period would be beneficial to fully assess the long‐term outcomes. Third, the definition of sarcopenia might not be accurate in every case. According to The European Working Group on Sarcopenia in Older People, the evaluation of both low muscle mass and function is recommended for sarcopenia diagnosis.^7^ However, due to the retrospective design of the study, the evaluation of muscle function, such as handgrip strength, proved challenging. Thus, further studies are warranted to accurately assess the association between preoperative changes in skeletal muscle mass and the prognosis of patients with PHC.

In conclusion, we demonstrated that preoperative skeletal muscle wasting in patients with PHC had a negative effect on survival outcomes.

## CONFLICT OF INTEREST

Yuko Kitagawa reports grants and personal fees from Chugai Pharmaceutical Co.; Ltd; Taiho Pharmaceutical Co., Ltd; Asahi Kasei Pharma Corporation; Otsuka Pharmaceutical Factory Inc.; Nippon Covidien Inc.; Ono Pharmaceutical Co., Ltd.; and Kaken Pharmaceutical Co., Ltd, grants from Takeda Pharmaceutical Co., Ltd.; Yakult Honsha Co. Ltd.; Otsuka Pharmaceutical Co., Ltd.; Tsumura & Co.; Kyouwa Hakkou Kirin Co., Ltd.; Sumitomo Pharma Co., Ltd.; EA Pharma Co., Ltd.; Astellas Pharma Inc.; Toyama Chemical Co., Ltd; Medicon Inc.; Eisai Co., Ltd.; Teijin Pharma Ltd.; and Nihon Pharmaceutical Co., Ltd., and personal fees from Shionogi & Co., Ltd.; Ethicon, Inc.; Olympus Corporation; Bristol‐Myers Squibb K.K.; AstraZeneca K.K.; MSD K.K.; Smith & Nephew KK; ASKA Pharmaceutical Co., Ltd.; Miyarisa Pharmaceutical Co. Ltd.; Toray Industries, Inc.; Daiichi Sankyo Company, Ltd.; Chugai Foundation for Innovative Drug; Discovery Science; and Nippon Kayaku Co., Ltd., outside the submitted work. The funding source had no role in the design, practice, or analysis of this study. Koki Hayashi, Yuta Abe, Minoru Kitago, Hiroshi Yagi, Yasushi Hasegawa, Shutaro Hori, Masahiro Tanaka, and Yutaka Nakano have no conflicts of interest or financial ties to disclose. Yuko Kitagawa is a current chief editor of *Annals of Gastroenterological Surgery* and none of the other authors of this article is a current Editor or Editorial Board Members of Annals of Gastroenterological Surgery. All authors' meets the authorship criteria and all authors are in agreement with the content of the article.

## ETHICS STATEMENTS

Approval of the research protocol: This study was approved by the Ethics Committee of the Keio University School of Medicine (Approval number: 20120443).

Informed Consent: The opt‐out method to obtain patient consent was utilized.

Registry and the Registration No. of the study/Trial: N/A.

Animal Studies: N/A.
